# COVID‐19 and the Canadian cattle/beef sector: A second look

**DOI:** 10.1111/cjag.12277

**Published:** 2021-03-30

**Authors:** James Rude

**Affiliations:** ^1^ Department of Resource Economics and Environmental Sociology University of Alberta Edmonton Alberta Canada

**Keywords:** beef supply chain, COVID‐19

## Abstract

After a year of adjusting to the shocks associated with COVID‐19 the Canadian cattle and beef sector faces a relatively optimistic future. This note examines the past year for this supply chain from the perspective of the consumer up to the cow‐calf producer by considering consumer reactions, labor market constraints, and supply responses. In the second quarter of 2020, the sector faced a significant challenge with continent wide shutdowns of beef packers reducing the U.S. beef supply by one‐third and Canadian beef slaughter by almost 60%. These shutdowns resulted in a sharp divergence between wholesale beef prices, which more than doubled, and fed steer prices, which declined by one third. Despite these dramatic shocks, the sector has returned to near normal conditions with prices and production levels similar to those observed prior to the pandemic. The near term prospects for 2021 are very similar to the current market situation.

## Résumé

Après une année d'adaptation aux chocs associés à la COVID‐19, le secteur bovin canadien fait face à l'avenir avec un relatif optimiste. Cette note examine l'année écoulée pour cette chaîne d'approvisionnement du point de vue du consommateur jusqu'au producteur de vache‐veau en tenant compte des réactions des consommateurs, des contraintes du marché du travail et des réponses de l'offre. Au deuxième trimestre de 2020, le secteur a été confronté à un défi de taille avec les fermetures à l'échelle du continent des abattoirs de bœuf, réduisant l'offre de bœuf aux États‐Unis d'un tiers et l'abattage de bœuf canadien de presque 60%. Ces fermetures ont entraîné une forte divergence entre les prix de gros de la viande bovine, qui ont plus que doublé, et les prix à la ferme, qui ont diminué d'un tiers. Malgré ces chocs dramatiques, le secteur est revenu à des conditions quasi normales avec des prix et des niveaux de production similaires à ceux observés avant la pandémie. Les perspectives à court terme pour 2021 sont très similaires à la situation actuelle du marché. Mots clés: COVID‐19, chaîne d'approvisionnement en viande bovine.

In March of 2020 when the effects of COVID‐19, and the associated shutdowns first started to be manifest across North America, there were immediate concerns about the integrity of the food supply chain. The first agri‐food sectors to be impacted by quarantines were at the consumer level with a massive redistribution of food between the hotel, restaurant, and institutional (HRI) sector and the grocery sector. It is relevant that grocery items that received significant attention—aside from toilet paper—included red meats (Lusk et al., 2021). Fortunately, meat shortages were minor, with a localized problem in some U.S. states and there never was a significant problem in Canada. As the impacts of the pandemic spread throughout the economy, COVID‐19 outbreaks spread across large North American regionalized beef packing plants. The outbreaks caused serious health concerns and spurred government interventions to protect worker safety as well as to provide set‐aside assistance to hold back slaughter cattle. The shutdown/slowdowns resulted in an escalation of wholesale prices while depressing farm‐level slaughter cattle prices. The divergence in these prices is something that was predicted by agricultural economists, yet this divergence was met with surprise (and possible overreaction) by U.S. policymakers and other commentators (Lusk et al., 2021). As the summer progressed so did calls from the general public to restructure the beef packing sector to be more resilient to shocks such as COVID‐19. Into the fall of 2020, public attention shifted away from bottlenecks in the meat supply chain to potentially higher food prices driven by higher overall commodity prices (Hume et al., 2021).

So, the natural question to ask, given that the 2020 CJAE special issue on COVID‐19 (Ker & Cardwell, 2020) was drafted at the effective start of the pandemic, is how well did I predict the events that subsequently unfolded? Given a year's worth of experience what are the surprises and what lessons can be learned? Unlike other livestock commodities beef's supply chain is protracted, more complex, and has less integration across sectors (Martinez et al., 2021). With a lag that extends up to two years before calves are marketed as slaughter cattle, the impacts of the initial demand side shock (driven by an almost 40% decline in real GDP for the second quarter) and subsequent packer shutdowns have not yet worked their way through the system back to cow calf investment/retention decisions. While large declines in quarterly real incomes were reported across North America, it is difficult to determine the income effect, if any, with respect to beef consumption because of a plethora of offsetting government programs. Therefore, it is more difficult to trace the secondary impacts of COVID‐19 up the beef/cattle supply chain.

I will discuss the impacts of the pandemic on the cattle/beef supply chain starting from the retail sector and working backwards to the primary producer. As in Rude (2020), most of the discussion will be descriptive, but now I have the benefit of hindsight and a more extensive body of literature to draw on.

## 1 RETAIL BEHAVIOR

The impacts of COVID‐19 for the beef consumer became known early in the pandemic. Business shutdowns and quarantine restrictions virtually closed down the HRI sector, so beef had to be diverted to grocery stores. The movement of beef products between sectors was not without complications because of differences in distributional logistics, package size, and meat qualities.^1^ However, over time there were no further adverse impacts beyond the surprises generated by the initial shocks.

While individual grocery shoppers tended to purchase cheaper cuts and hamburger, sales of more expensive restaurant cuts (prime rib) declined. Stock‐outs at supermarkets initially posed a problem for frozen storable products, but less so for fresh meats. Some of the difficulties in adjustment can be attributed to in‐store logistics, but also to a system built around just‐in‐time manufacturing and unexpected spikes in demand (Goddard, 2020).

During April and May, some consumer hoarding occurred in anticipation of restricted mobility and limited supplies. Meat shortages were limited with some localized problems in the United States (e.g., hamburger shortages in Wendy's) but Canada never faced shortages and two‐way trade between Canada and the United States smoothed supplies. Certainly, any shortages were not a result of a shortage of animals, or disruptions along the distribution network. Labor shortages and physical shutdowns in the beef‐packing sector were the major cause of disruptions in the sector. Retail prices are closely related to wholesale prices, so when wholesale prices increased (see below) retail prices also increased but by a lower percentage (30% in the United States [BLS, 2020] and 15%–20% in Canada [Statistics Canada, 2020). Since August, retail prices have returned to pre‐COVID‐19 levels.

## 2 PROCESSING SECTOR

**FIGURE 1:**
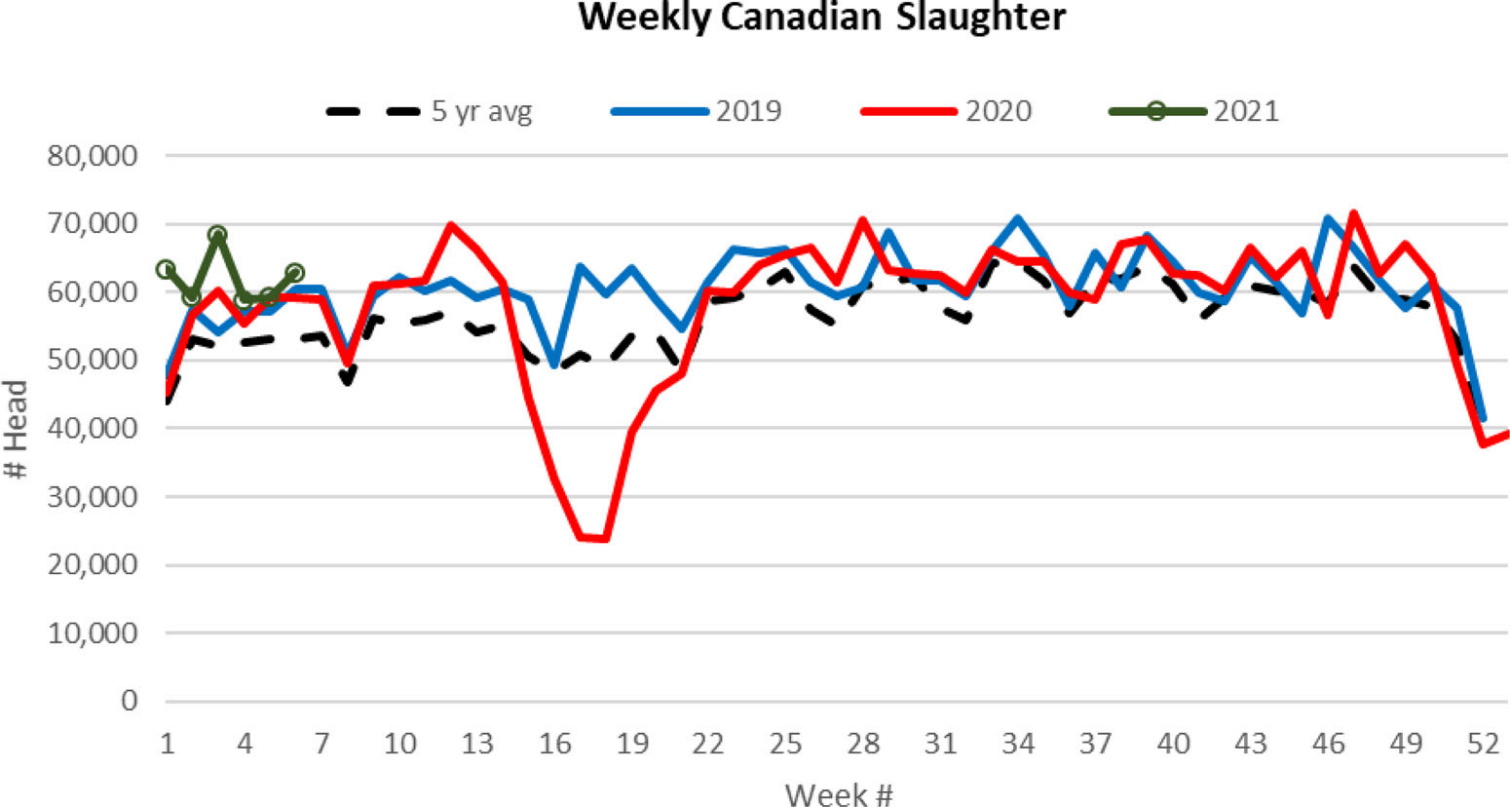
Weekly cattle slaughter *Source*: Canfax (2020b)

Across North America, a series of meatpacker shutdowns and slowdowns occurred extending from late March to mid‐June. At its peak in late April, U.S. weekly beef production declined by one‐third (USDA AMS, 2020). In terms of Canadian beef packers, Cargill's High River Alberta plant was responsible for the single largest COVID‐19 outbreak (almost 900 employees were infected) for North American meat packers. At the JBS Brooks Alberta plant, more than 500 of the 2,600 employees contracted COVID‐19 (Ross, 2020). As a result, between April and the middle of May the Cargill plant had to close and the JBS plant faced significant slowdowns. Canadian beef slaughter declined by 54% between the first quarter of 2020 and the peak disruption in mid‐May (see Figure 1). Certainly, the degree of the meat packing disruptions was the most unexpected impact on the beef sector in 2020 from COVID‐19 in 2020.

Constrained labor availability forced plants to make major adjustments that included the introduction of safety screens, barriers, physical‐distancing protocols, restrictions on car‐pooling, and other measures to try to prevent additional COVID‐19 outbreaks. All of these adjustments affect line speed and capacity utilization in a system where the incentives are for packing plants to operate at as high of a capacity as possible.

The Cargill and JBS Alberta plants were constructed in the late 1980s and they are nearing the end of their economic lives (Rude, 2021). Decisions will have to be made about renovations, new construction, and location of facilities to meet North American product mandates. Across North America, public arguments have been made that smaller, more regionally dispersed plants would have reduced the probability of the spread of COVID‐19 and resulted in a more secure meat supply. However, this may or may not be true. There are two opposing effects determining the overall potential impact for the spread of the virus. First, regardless of scale, processing takes place in a refrigerated assembly line with close worker contact, and poor air circulation (The Meating Place, 2020). So, reducing the scale of the packing plants is unlikely to reduce the spread of the virus. The second effect relates to regional diversification and the probability that the virus is not spread equally across regions (Lusk, 2020). There is no empirical evidence about which of these opposing effects dominates and whether smaller more regionally distributed packing plants would be less likely to spread COVID‐19 (Rude, 2021).

### 2.1 Economics of smaller more regionally distributed packing plants

This section is drawn from Rude (2021) where I argue that reorganizing beef packing to favor regionally distributed plants is not feasible. Incumbent firms have significant advantages relative to potential entrants in the form of scale economies and the ability to procure animals from concentrated fed cattle supplies. Mandated changes to protect the health of workers will add even more regulatory fixed costs that must be spread over greater volumes of meat production. This will favor larger producers who are able to achieve lower unit costs. Other changes on the horizon that are likely to be adopted earlier because of COVID‐19 include the adoption of robotic meat cutting. However, these innovations are most likely to be adopted by current large‐scale packers who have already made large investments in these innovations (see Rude (2021) for more details). These innovations would also put potential small‐scale entrants at a disadvantage.^2^

The strongest counter‐argument, against promoting small and medium sized packers, is that there are natural forces that limit slaughter to regions where the livestock is raised and that significant scale advantages limit the total number of slaughter plants across North America. Over the last four decades, the North American beef‐packing sector has undergone considerable consolidation (Azzam, 1998; MacLachlan, 2001). The trend towards fewer and larger plants was driven by the enhanced economic efficiency and cost management associated with operating larger firms. Ward (2010) and MacDonald et al. (2000) estimated that minimum efficient scale for a U.S. beef packing plant was in the 1 to 1.1 million animals per year range. Cargill in High River and JBS in Brooks each process over 1.1 million animals annually (Canfax, 2020a). Koontz (2020) identified the typical U.S. commercial slaughter plant as annually processing between 1.3 and 1.8 million animals. MacDonald et al. (2000) found that processing costs for large meatpacking plants were 12% lower than for medium sized plants and 25% to 40% lower than for small plants.

The potential for construction of small and medium sized plants is limited by construction costs (Rude (2021) suggests between $31 million and $47 million), fierce competition from established large‐scale competitors to procure animals, and overall operating cost disadvantages. Therefore, small and medium sized packers cannot compete on a cost basis with larger operations. They will have to compete in niche markets through product differentiation where they attract new buyers who are willing to pay premium prices that cover the extra costs associated with these types of plants. COVID‐19 may have changed consumer behavior in favor of local food because some consumers believe, in part, that local food supplies are more resilient and reliable in uncertain markets (Hobbs, 2020). Interest has peaked for direct farm‐to‐consumer sales. The province of Alberta has changed legislation so that the meat from animals killed by mobile butchers and farmers licensed to carry out uninspected slaughter can be sold directly by farmers to the public. Nonetheless, many consumers will continue to demand low priced sources of protein and only the current system of large‐scale regional beef packers can provide beef at current prices.

Regardless of whether the current system of beef packing continues or if more small and medium sized packers are built, adjustments will have to be made going forward. While commercial meatpacking plants are physically configured to optimize efficiency, they are not configured to account for risks to human health from working in close proximity. The usual definition of resilience relates to the capacity to recover quickly from difficulties by being adaptable and nimble. Future adjustments to improve stress‐time processing resilience probably will include slower processing line speeds, and will reduce capacity utilization. One innovation that would minimize these additional costs is the rapid adoption of meat‐cutting robotics to protect human health and reduce the frequency of shutdowns (see Rude, 2021). Nonetheless, there is a trade‐off between efficiencies and general stress‐time resilience (Grandin, 2020).

## 3 FEEDLOT AND COW‐CALF SECTORS

Although labor shortages can affect feedlot and cow‐calf operations through a network of auction barns, feed mills and associated transportation services, no significant labor disruptions occurred throughout this network over the past year. While these sectors were not directly affected by labor disruptions, they were nonetheless impacted through changing prices. Since the feedlot sector is the closest in the supply chain to beef packers, it was the first link affected.

**FIGURE 2:**
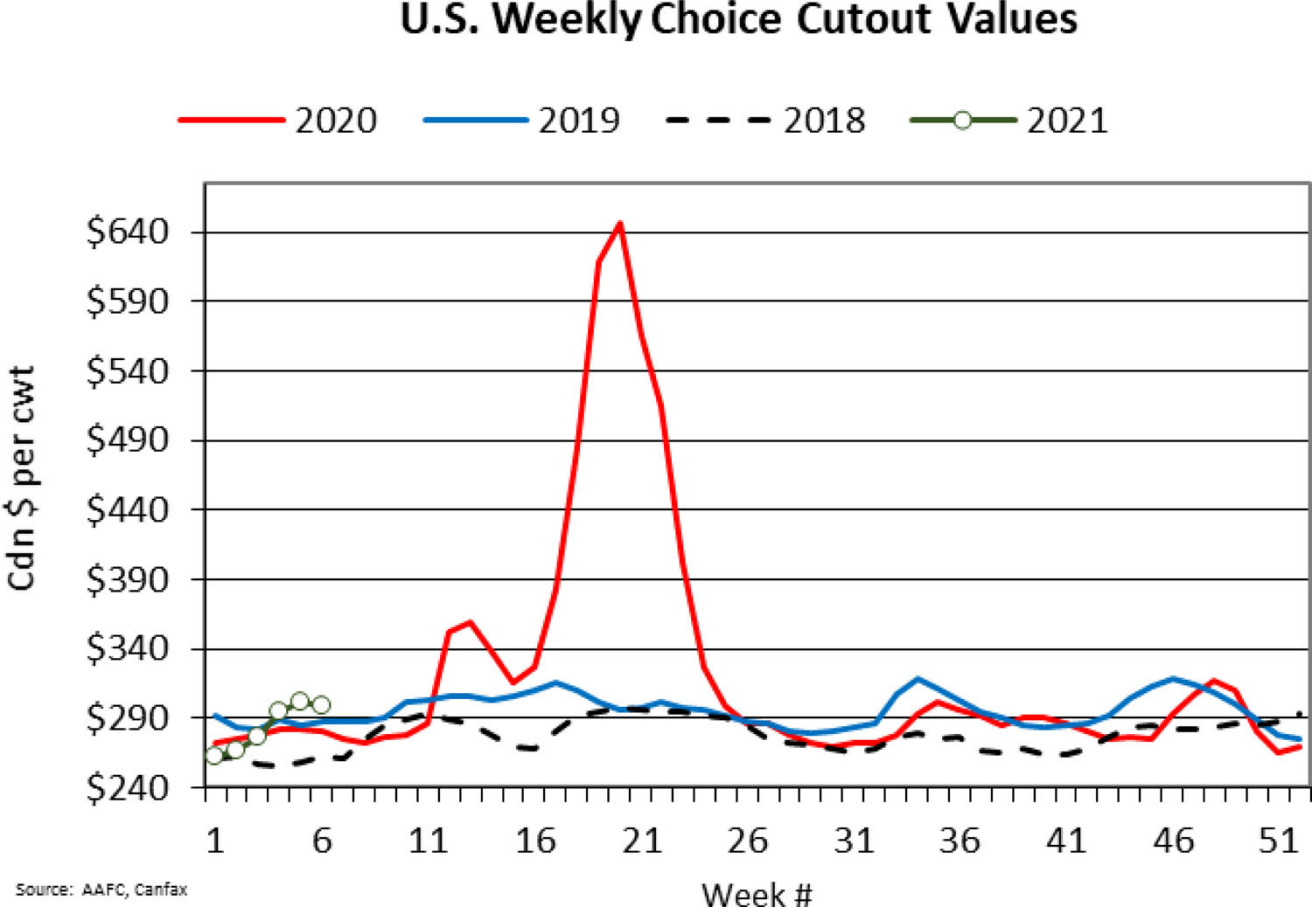
Wholesale prices of beef *Source*: Canfax (2020b)

**FIGURE 3:**
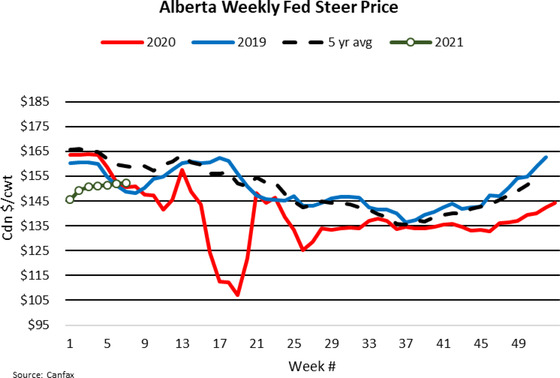
Weekly fed livestock price *Source*: Canfax (2020b)

Given reduced slaughter across North America, there was decreased demand for fed livestock and a corresponding decline in the wholesale supply of meat. The workings of the market ensured that wholesale prices increased and that farm prices declined (Lusk et al., 2020). At the peak of the crisis, wholesale boxed‐beef prices (see Figure 2) more than doubled (127%) from the prices in the first quarter of 2020 because of the COVID‐19 induced production slowdowns. The slaughter backlog led to reduced demand for fed cattle resulting in a 36% lower Alberta fed steer price between the first quarter of 2020 and the price in late May (see Figure 3).

At its peak in late April, the backlog of western Canadian cattle waiting to be slaughtered was 133,000 head (Grant, 2020). Because of the backlog, feedlots faced higher input costs where rations had to be adjusted to hold cattle on feed longer while at the same time trying to minimize weight gain. Processors penalize producers for animals that are heavier than average. Over time, the backlog has been reduced to manageable proportions (Grant, 2020).

The cattle industry called for government money to assist with financing maintenance rations and other associated opportunity costs. Together the federal and provincial governments provided a set‐aside program ($125 million), through the *Canadian Agricultural Policy* framework program *AgriRecovery* 3 that provided per head payments to cover the costs of maintaining market‐ready cattle until the inventory backlog could be cleared (Western Stock Growers Association, 2020).^4^ Although most government COVID‐19 programs were generally available to all of agriculture or industry more broadly, this is an example of a targeted, sector specific program.

Cow‐calf producers are at the end of the supply chain, with a lag of two years before calves are marketed as slaughter cattle. Over time, the cow‐calf sector may be more exposed to prolonged COVID‐19 disruptions because losses in other sectors accrue to ranchers by way of lower prices (Martinez et al., 2021). The impacts associated with COVID‐19 were not expected until at least late in 2020. The Canadian Cattlemen's Association stated, “(f)ew lasting effects trickled down to the non‐fed and feeder markets. Cow‐calf profitability has been supported by outstanding demand for beef at retail this year and lower feed costs” (Canadian Cattlemen Beef Magazine, 2020). Over the first three quarters of 2020, cull cow slaughter was down by 21% in western Canada and 5% in the east. Nonetheless, cull (D1‐D2) cow prices were steady through 2020 (Canadian Cattlemen Beef Magazine, 2020). An ongoing concern is that the Canadian cattle inventory has been in decline, by 25%, since 2005 and it continued to decline in 2020. Between 2019 and 2020 the breeding‐cow herd declined by 1.4% and total cattle inventories declined by 0.5%. The decline in total cattle numbers happened despite processing disruptions delaying fed cattle slaughter (Canadian Cattlemen Beef Magazine, 2020). In order for Canadian cattle producers to take advantage of potential export growth the decline in the Canadian breeding inventory has to stop. While the herd continued to decline over the last year, the rate of decline did not significantly increase from the historic trend as a result of COVID‐19.

## 4 INTERNATIONAL TRADE

Early in 2020, the potential for offshore exports of beef was particularly promising because *African Swine Fever* had decimated hog herds in China, and world markets for animal proteins were becoming very tight. With the onset of COVID‐19 restrictions, the industry's concern was that logistical frictions, labor shortages for inspectors and customs agents, and interruptions for trucking would contribute to border thickening. Border thickening involves regulations and costs that result in slowdowns and difficulties for moving people and goods across international borders. Border thickening can also result from political uncertainties. On March 18, 2020, the Government of Canada announced the closure of the U.S.–Canada border to nonessential travel (tourism and recreation). At the same time, assurances were given that travel restrictions and closures will not negatively affect the flow of trade. To date, international trade has flowed smoothly with relatively few frictions, so there were few unexpected trade impacts from COVID‐19 in 2020.

For the period January to July 2020 beef exports were down 9% in volume and 2% in value, relative to the same period in 2019 (Canadian Cattlemen Beef Magazine, 2020). Exports were affected by domestic supply disruptions and negative demand shocks in several importing countries. By year‐end, in value terms, 2020 Canadian beef exports were 1.25% greater than in 2019, while in volume terms exports were down by 3.1% (AAFC, 2021).

As markets begin to stabilize after the initial shock of COVID‐19, offshore beef and other meat demand should help the Canadian beef cattle sector to recover from the initial shock; however, the path forward is obscured with uncertainties. The full impact of the second wave of COVID‐19 infections is not yet known despite the growing distribution of vaccines and the potential for herd immunity. Although some pork packers have recently been forced to shut down by viral infections, we cannot tell if there will be similar shutdowns for beef packing. Even without further disruptions, the impact for Canadian exports is uncertain. Going into the future the impact of the pandemic on domestic and foreign incomes will determine the future of exports.

Although there are a wide variety of types of economic models that attempt to explain trade flows, the conventional set of explanatory variables typically include real exchange rates and national incomes. Export demand can involve long lags with respect to income shocks. The challenge is not just to determine the appropriate lag length, but also to determine the depth of the income reduction. Although developed countries have experienced sharp income declines, a plethora of government programs have helped to offset much of the income decline. Therefore, it is difficult to determine the effective decline in income and thus the impact on beef consumption. Since beef is one of the commodities with the highest income elasticities, import demand elasticities will be even larger, and the potential adverse trade effects are correspondingly larger.

In March of 2020, the Canadian/U.S. exchange rate was expected to depreciate. However, going into the second year of the pandemic the prospects for the Canadian dollar are mixed (RBC Economics, 2021). On one hand the Canadian dollar is expected to follow a path of overall commodity price increases so that there is potential for a stronger Canadian dollar (Hume et al., 2021). On the other hand, a strong U.S. economy as it emerges from the COVID‐19 recession would lead to a weaker Canadian dollar. An appreciation will make Canada's beef exports less competitive, while at the same time, the cost of imported inputs should decline.

The potential for offshore exports of Canadian beef is still promising. Nonetheless, there are protectionist pressures associated with COVID‐19, as a result of a quest for food security and international cooperation, which is always in a “delicate balance” (Kerr, 2020). Even with change in the U.S. administration, protectionism has not disappeared. Despite threats of protectionism two‐way beef trade allowed both Canadian and U.S. consumers to procure adequate supplies of beef during the worst of the 2020 beef packing plant shutdowns (Mallory, 2021).

## 5 LESSONS LEARNED: THE EXPECTED, THE UNEXPECTED, AND WAY FORWARD

With a year of COVID‐19 experience behind us, we have learned a few lessons about adapting to a major disruption to agricultural markets, but in many other ways there are some familiar uncertainties going forward as when the first version of this CJAE special issue (Ker & Cardwell, 2020) was drafted. Across the agri‐food sector, there has been remarkably little disruption notwithstanding the size of the initial labor and income shocks. The sector has adjusted remarkably well. Despite the numerous links in the agri‐food supply chain, labor disruptions have been limited to only a few sectors (mostly meatpacking). While disruptions in the HRI sector were typically associated with government‐mandated shutdowns, the role of government restrictions had much less impact on the rest of the beef cattle supply chain.

The Canadian cattle industry entered 2020, and the COVID‐19 disruption, with a breeding herd that had been declining for 15 years, a concentrated and aging processing sector, concerns about the rise of meat substitutes, and pressures for the industry to reduce its carbon footprint. Yet, the prospects for Canadian beef exports remain as rosy today as they were at beginning of 2020. However, to remain competitive the Canadian industry must overcome serious challenges. Beef supply chains will always be vulnerable to potential disruptions.^5^ Disruptions to processing capacity harms primary producers, meat processors, consumers, and our trading partners. In response to a call for improved stress‐time resilience, there have been calls from a number of quarters for the construction of small and medium sized packing plants. Additional health and safety regulations add to fixed costs that in turn increase scale economies making smaller plants even less competitive. Furthermore, additional slack (e.g., slower line speeds) may be introduced, through regulatory reform, into the supply chain which ultimately increases the cost of beef.

So, what was expected? Although Rude (2020) used a model of the North American beef sector, the purpose of the exercise was never to provide nuanced predictions, but rather to define guideposts for possible outcomes. My prediction for the second quarter price of fed steers came remarkably close to what happened. However, while the primary shock that drove my results was income based (and which depressed all prices) while the dominant shock driving cattle prices was a major slowdown/shutdown by North American beef packers (which increased wholesale and retail prices).^6^ Consequently, the biggest, unexpected event for 2020 beef markets was the impact of packer shutdowns across North America and the fragility of the beef‐processing sector. The way forward involves difficult trade‐offs to make sure that the beef supply chain does not face future disruptions of a similar magnitude to COVID‐19. Rude (2021) discusses alternative measures (including robotics) to make the beef processing sector more resilient. There is no one‐size fits‐all solution to improve resilience. Rather it depends on a learning process from the COVID‐19 experience and adherence to risk management best practices.

## Footnotes

1 Sections of this paper are drawn from my contributions to Weersink et al. (2021).

2 Some industry stakeholders have asked for relaxed inspection standards for smaller packers (e.g., to allow interprovincial trade for provincially inspected meat). These reforms could possibly reduce some fixed cost for smaller plants and thereby reduce their unit costs.

3 The Fed Cattle Feed Assistance program ran from May 1 to June 30 and was followed a by bid set‐aside process which gradually expended the remaining funds over the course of 2020. The average bid for the bid‐set‐aside was $1.73 per head per day (Real Agriculture, 2020). Increasing demand by packers quickly eliminated the need for the program. On the U.S. side of the border producers were offered per head payments across the board at $55 dollar per head (USDA, 2020).

4
*AgriRecovery* costs are shared 60% by the federal government and 40% by the provinces. However, most of set‐aside money was distributed to Alberta feedlots.

5 Relative to the other types of disruptions that could have occurred—for example, reportable diseases such as *Foot and Mouth Disease* in cattle—the impacts of COVID‐19 have so far been relatively minor. This type of disease would have shut the border to exports, disrupted interprovincial trade, and lead to drastic reductions in livestock herds and the associated adjustment costs.

6 My model included both income shocks and idling of processing capacity. In terms of exogenous shocks the income effects dominated the slowing of processing capacity.
